# Upgrading Mitochondria-Targeting Peptide-Based Nanocomplexes for Zebrafish In Vivo Compatibility Assays

**DOI:** 10.3390/pharmaceutics16070961

**Published:** 2024-07-20

**Authors:** Rúben Faria, Eric Vivès, Prisca Boisguérin, Simon Descamps, Ângela Sousa, Diana Costa

**Affiliations:** 1 CICS-UBI—Health Sciences Research Centre, University of Beira Interior, 6201-001 Covilhã, Portugal; ruben.faria@ubi.pt (R.F.); angela@fcsaude.ubi.pt (Â.S.); 2 PhyMedExp, University of Montpellier, INSERM, CNRS, 34295 Montpellier, France; eric.vives@umontpellier.fr (E.V.); prisca.boisguerin@inserm.fr (P.B.); 3 CRBM-CNRS, Cell Biology Research of Montpellier, UMR5237, 34293 Montpellier, France

**Keywords:** cell-penetrating peptides, hemocompatibility, in vivo toxicity, mitochondria-targeting, peptide-based nanoparticles, Zebrafish embryo model

## Abstract

The lack of effective delivery systems has slowed the development of mitochondrial gene therapy. Delivery systems based on cell-penetrating peptides (CPPs) like the WRAP (tryptophan and arginine-rich peptide) family conjugated with a mitochondrial targeting sequence (MTS) have emerged as adequate carriers to mediate gene expression into the mitochondria. In this work, we performed the PEGylation of WRAP/pDNA nanocomplexes and compared them with previously analyzed nanocomplexes such as (KH)_9_/pDNA and CpMTP/pDNA. All nanocomplexes exhibited nearly homogeneous sizes between 100 and 350 nm in different environments. The developed complexes were biocompatible and hemocompatible to both human astrocytes and lung smooth muscle cells, ensuring in vivo safety. The nanocomplexes displayed mitochondria targeting ability, as through transfection they preferentially accumulate into the mitochondria of astrocytes and muscle cells to the detriment of cytosol and lysosomes. Moreover, the transfection of these cells with MTS–CPP/pDNA complexes produced significant levels of mitochondrial protein ND1, highlighting their efficient role as gene delivery carriers toward mitochondria. The positive obtained data pave the way for in vivo research. Using confocal microscopy, the cellular internalization capacity of these nanocomplexes in the zebrafish embryo model was assessed. The peptide-based nanocomplexes were easily internalized into zebrafish embryos, do not cause harmful or toxic effects, and do not affect zebrafish’s normal development and growth. These promising results indicate that MTS–CPP complexes are stable nanosystems capable of internalizing in vivo models and do not present associated toxicity. This work, even at an early stage, offers good prospects for continued in vivo zebrafish research to evaluate the performance of nanocomplexes for mitochondrial gene therapy.

## 1. Introduction

Mitochondria are essential for the normal functioning of eukaryotic cells. These small organelles are involved in diverse cellular processes such as signaling, growth, and regulation of cell division and ion homeostasis or apoptosis mechanisms, among others [1,2]. However, the highlight is the role they play in energy production through the process of mitochondrial oxidative phosphorylation (OXPHOS). Inside mitochondria, energy is produced in the form of adenosine triphosphate (ATP) molecules [3,4,5]. This organelle is responsible for producing more than 90% of all energy produced within cells and it is considered the cell’s engine [6]. In addition to their crucial importance in normal cellular functioning, mitochondria have a particular characteristic that they share with the nucleus: they have their own genome [7]. The mitochondrial genome (mtDNA) is composed of circular DNA molecules, which contain 37 genes encoding 13 proteins that act as subunits of the OXPHOS enzymatic complexes, 22 transport RNA (tRNA) molecules, and 2 ribosomal RNA (rRNA) molecules [8,9].

Mutations in mtDNA are widespread compared to mutations in the nuclear genome. This is due to the much higher number of mtDNA molecules per cell than nuclear DNA and the absence of an effective repair mechanism and protective histones [10,11]. Mutations in mtDNA can induce a wide range of pathologies: neurodegenerative diseases (Alzheimer’s, Parkinson’s, and Amyotrophic Lateral Sclerosis (ALS)), metabolic dysfunctions (diabetes), hereditary diseases (Leber hereditary optic neuropathy (LHON), Huntington’s disease), and various types of cancer [12,13,14,15]. Therapeutics currently available on the market only slow the progression of these diseases and do not provide an effective treatment [16,17]. Thus, there is a need to develop new therapeutic approaches. Since these diseases arise from gene alterations, mitochondrial gene therapy appears as a very promising/effective strategy [18].

Mitochondrial gene therapy has as its principle the replacement of mitochondrial genes to restore the normal function of the target gene [19,20]. To achieve this, it is necessary to use a delivery system that is capable of transporting and protecting the therapeutic genetic material and that delivers the mitochondrial gene directly to the mitochondria, enabling its expression [21,22]. One of the most explored genetic material delivery systems in recent years has been cell-penetrating peptides (CPPs). CPPs are peptides with fewer than 30 amino acids that have hydrophobic and hydrophilic domains, which confers the ability to conjugate with genetic material, forming nanoparticles capable of transfecting and internalizing into cells [23,24,25,26]. To confer specificity for mitochondria—a key step in mitochondrial gene therapy—some strategies have been explored, including the utilization of the 12-residue partial pre-sequence of yeast cytochrome c oxidase subunit IV (MLSLRQSIRFFK) [27,28,29].

Zebrafish (Danio rerio) is a vertebrate animal model increasingly applied in scientific research. Due to its characteristics, its application has been explored in the most diverse areas, from biomedicine and biotechnology to environmental science [30,31,32,33]. The main advantages of zebrafish (ZF) compared to other vertebrate models are the fact that it is an animal of small size, thus easy to maintain at low costs; its reproduction rate is high (each female releases an average of between 50 and 300 fertilized eggs); one-cell-stage fertilized eggs are easily genetically manipulated; its embryos and larvae are transparent; embryonic development occurs within 24 h and formation of the heart, intestine, and blood vessels within 48 h of fertilization. Furthermore, the ZF genome is approximately 70% similar to the human genome and its physiological processes including the development of the cardiovascular, nervous, and digestive systems are similar to those in humans [32,34]. Consequently, the ZF model has been widely considered in pre-clinical trials and the most varied toxicological studies [35,36,37].

In this work, our goal was to add PEG (polyethylene glycol) to the MTS–WRAP (tryptophan and arginine-rich peptide) delivery systems, determine their properties, evaluate their mitochondria targeting ability, and, ultimately, evaluate them in an in vivo model, namely their internalization capacity and toxicity in ZF embryos. The PEG-free MTS–WRAP–based complexes were tested in vitro in previous publications by our team, where we demonstrated excellent results from our physicochemical and morphological characterization to the production of the target mitochondrial protein [29,38]. 

The PEG–WRAP conjugate was added to the formulation of MTS–WRAP nanocomplexes, using three different percentages (5%, 10%, and 20%), and compared to previously analyzed non-PEGylated nanocomplexes such as (KH)_9_/pDNA and CpMTP/pDNA. PEG is a biocompatible polymer that can be linear or branched, whose main characteristics are that it increases the half-life of a therapeutic agent, increases the circulation time of the biomolecule to which it is conjugated, increases its hydrophilicity, and decreases the probability of agglomeration [39,40]. PEGylation of nanoparticles has been shown to increase their stability and consequently reduce the toxicity of delivery systems. PEGylation also reduces immunogenicity, increases the biological half-life of these therapeutic agents, and enables greater efficiency with smaller doses [41,42,43]. The developed PEG–MTS–WRAP nanocomplexes were demonstrated to be stable over time (up to 7 days after formulation), with sizes suitable for cellular transfection. Moreover, the complexes were biocompatible and hemocompatible and displayed the ability to transfect and internalize into ZF embryos, without causing toxicity. The results evidenced in this report indicate that the conceived peptide-based delivery systems possess a set of favorable assets being a valid option for mitochondrial gene therapy implementation, and should be considered for future in vivo studies with a view to potential clinical translation.

## 2. Materials and Methods

### 2.1. Materials

Milli-Q water, 1-phenyl 2-thiourea (PTU), and Tricaine powder were obtained from Sigma-Aldrich (Waltham, MA USA). ULYSIS Nucleic Acid Labeling Kit was obtained from Thermo Fisher Scientific Inc. (Waltham, MA USA). All peptides were synthesized using a LibertyBlue™ Microwave Peptide Synthesizer (CEM Corporation, Stallings, NC, USA) with an additional Discover™ module (CEM Corporation, Stallings, NC, USA) combining microwave energy at 2450 MHz to the Fmoc/tert-butyl (tBu) strategy. Peptide identity and purity were checked by LC-MS (Waters, Saint-Quentin-en-Yvelines, France). 3-(4,5-Dimethylthiazol-2-yl)-2,5-diphenyltetrazolium bromide (MTT), Triton X-100, and fluorescein isothiocyanate (FITC), isomer 1, were obtained from Sigma-Aldrich Chemicals (St. Louis, MO, USA). The plasmid pCAG-GFP-ND1 (5.4 kbp) (pND1) was developed by our research group through the cloning of the mitochondrial NADH dehydrogenase 1 protein-encoded gene (mtND1) in *Escherichia coli*. The details concerning ND1 gene cloning and plasmid production can be consulted elsewhere [44]. Human astrocyte cell line (HA1800), lung smooth muscle cells, normal, human (PCS-130-010), and human embryonic kidney (HEK293T) cells were obtained from the American Type Culture Collection (ATCC, Manassas, VA, USA).

### 2.2. Methods

#### 2.2.1. Nanoparticle Formulation

The formulation of WRAP/pND1, MTS–WRAP/pND1, (KH)_9_/pND1, MTS–(KH)_9_/pND1, and CpMTP/pND1 nanoparticles was carried out as previously described [29,38]. The PEG nanoparticles were formulated using the same protocol mentioned above, however, the PEG-WRAP peptide was added in 3 different percentages (5%, 10%, and 20% of the total peptide amount) to formulate the respective vectors. All nanoparticles were formulated considering a nitrogen-to-phosphate groups ratio (N/P) of 5.

#### 2.2.2. Particle Size Measurements

The average size of the nanoparticles was determined by Dynamic Light Scattering (DLS) using a Zetasizer Nano ZS (Malvern Instruments, Malvern, UK) equipped with Malvern Zetasizer software v6.34. DLS using a He–Ne laser 633 nm with non-invasive backscatter optics (NIBS) was applied for size determination. All results were obtained from three independent measurements (three runs for each measurement at 25 °C).

#### 2.2.3. Cell Culture

HA1800 and HEK293T cells were cultured in Dulbecco’s modified Eagle’s medium (DMEM) containing 10% FBS. Primary lung smooth muscle cells were maintained in vascular cell basal medium (ATCC, PCS-100-030) supplemented with 5% heat-inactivated FBS, 5% L-glutamine, 0.5 mL penicillin–streptomycin–amphotericin B solution (penicillin 10 units/mL, streptomycin 10 μg/mL and amphotericin B 25 ng/mL), 5 ng/mL of basic-fibroblasts growth factor (b-FGF), 5 ng/mL epidermal growth factor (EGF), 50 μg/mL of ascorbic acid, and 10 ng/mL of insulin. All cell lines were incubated at 37 °C and 5% CO_2_ until ~80% of confluence was attained. Cells were sub-cultivated every 3 days to maintain their exponential growth and normal metabolism.

#### 2.2.4. Cytotoxicity Evaluation

The cytotoxicity of the developed MTS–CPP/pND1 systems was evaluated in human astrocyte cells and lung smooth muscle cells using the MTT (3-[4,5-dimethyl-thiazol-2-yl]-2,5-diphenyltetrazolium bromide) assay. The assay was performed in 96-well plates at a density of 1 × 10^4^ cells/well, where the cells were serum starved 12 h before incubation with the nanoparticles. The cells were incubated with the nanoparticles (0.1 µg of pND1 per well), and the transfection was stopped 6 h later by changing the cell medium to complete medium (with serum). After 24 and 48 h of incubation with the systems, cell viability was assessed by reducing the MTT. For this, 20 µL of MTT solution with a concentration of 2 mg/mL was added to each well for 2 h. After that, the medium was removed and 200 µL of DMSO was added to each well for 30 min with shaking to dissolve the formazan crystals. Absorbance was measured using a Benchmark Microplate Reader (BioRad, Vienna, Austria) at 570 nm. The medium without cells was set as zero absorbance and used for spectrophotometer calibration. Non-transfected and ethanol-treated cells were considered the positive and negative controls, respectively. The relative cell viability (%) related to control wells was calculated by [A]test/[A]control × 100, where [A]test is the absorbance of the test sample and [A]control is the absorbance of the positive control sample.

#### 2.2.5. Hemolysis Test

In the hemolysis test, fresh rat blood was used, which was previously collected and stored in heparinized tubes containing EDTA disodium salt. Subsequently, red blood cells (RBCs) were isolated through centrifugation (3000 rpm for 15 min at 4 °C). The RBCs were washed with 0.85% *w*/*v* NaCl solution until the solution became translucent. For the assay, the nanocomplexes (PEG–MTS–WRAP/pND1 and MTS–(KH)_9_/pND1) and RBCs were resuspended in PBS (pH 7.4). A PBS-based solution containing 3–5% RBCs was prepared. 900 µL of this solution was used for each condition, where 100 µL of each of the delivery systems under study was subsequently incubated. The negative control was performed by adding 100 µL of PBS (pH 7.4) to 900 µL of the RBC solution while the addition of 100 µL of Triton X-100 (1%) was considered for the positive control. Incubation took place for 1 h at 37 °C. After incubation, the samples were centrifuged at 8000 rpm for 20 min at 4 °C. The supernatant obtained was analyzed by measuring its absorbance at 576 nm in a UV-vis spectrophotometer. The percentage of hemolysis was calculated using the following formula:

(1) $$Hemolysis\left(\%\right)=\frac{Abs.Sample-Abs.Negative\;control}{Abs.Positive\;control-Abs.Negative\;control}\times 100$$

#### 2.2.6. FITC Plasmid Staining

Plasmid DNA was stained with FITC by mixing 2 µg of pDNA, 2 μL of FITC (in sterile anhydrous dimethyl sulfoxide, 50 mg/100 µL), and 81 μL of labeling buffer (0.1 M Sodium Tetraborate, pH 8.5). Samples were left in the dark, under stirring for 4 h at 25 °C. To stop the reaction, 2.5 volumes of 100% ethanol (212.5 μL) and one volume of 3 M NaCl (85 μL) were added. Samples with stained pDNA were precipitated at −20 °C overnight. On the following day, samples were centrifuged (10,000× *g*, at 4 °C) for 30 min. The pellet was recovered and washed with ethanol (75%) and used for the formation of PEG/MTS–peptide/pND1 complexes.

#### 2.2.7. Cellular Organelle-Associated FITC Fluorescence 

Human astrocytes and lung smooth muscle cells were cultured as described above. For cellular transfection, PEG/MTS–peptide/pND1-FITC (100 μL, pND1 = 1 µg) was added to each well. Untreated cells incubated with FITC and naked pND1 stained with FITC were used as controls. After 24 h, both cells were washed twice with PBS, and FITC-pND1 levels were measured in the transfected cells by fluorescence quantification. A fluorimeter plate reader was used to determine FITC fluorescence considering the excitation and emission wavelengths of 495 nm and 525 nm, respectively. For each cell line, the protein content of each well was measured with a bicinchoninic acid (BCA) protein assay kit (BCA1-1KT, Sigma Aldrich Chemicals, St. Louis, MO, USA). Fluorescence/microgram protein readings were then determined by averaging the background corrected fluorescence of triplicate wells and dividing by the protein content per well. After transfection of human astrocytes and lung smooth muscle cells with the developed MTS–CPP/pND1–FITC complexes, the Mitochondria Isolation Kit for Cultured Cells (#89874, Thermo Fisher Scientific Inc., Rockford, IL, USA) was applied to promote the separation of mitochondria from the cytosol. The experimental protocol provided by the manufacturer has been followed, as described elsewhere [44]. In another set of transfected astrocytes and muscle cells, with the developed MTS–peptide/pND1–FITC complexes, lysosomes were isolated by using the Lysosome Isolation kit (LYSOSO 1) according to a protocol available from the supplier and as previously presented in the literature [45]. The protein levels of each cellular organelle were determined using the BCA protein assay kit. The use of this kit aids in correcting for cell density differences between different sets of experiments. The fluorescence of FITC–pND1 in each organelle sample was normalized with the amount of protein and expressed as fluorescence/µg protein. 

#### 2.2.8. ND1 Protein Quantification

Mitochondrial ND1 protein levels were quantified in human astrocyte, lung smooth muscle cells using an ND1 ELISA kit (Biomatik, EKL54820, Wilmington, DE, USA), as fully described in a previous publication by our research team [38]. After 48 h of transfection with the systems under study, the cells were collected and lysed. The determination of ND1 protein content was performed according to the protocol provided by the manufacturer, which, and to summarize, includes the incubation of Reagent A for 1 h at 37 °C, followed by the addition of Reagent B. Then, an incubation was carried out with the TMB solution for 20 min at 37 °C, in the dark. Finally, the Stop solution was added to end the reaction. The ND1 levels were determined by absorbance at 450 nm.

#### 2.2.9. Zebrafish Breeding

Zebrafishes (*Danio rerio*) were raised and used according to standard laboratory protocols. Zebrafish care and use were performed in accordance with European Union guidelines for the handling of laboratory animals. All experiments were approved by the Comité d’Ethique pour l’Expérimentation Animale and the Direction Sanitaire et Vétérinaire de l’Hérault (Aquatic model facility, ZEFIX from CRBM C-34-172-39). Tg[fli1a:EGFP]y1 transgenic line was used with GFP-labelling of all endothelial cells [46]. ZF embryos were kept for 24 h in a dish (100 mm) in E3 medium (5 mM NaCl, 0.17 mM KCl, 0.33 mM CaCl_2_, 0.33 mM MgSO_4_, 0.01 mg/L Methylene Blue) in an incubator at 28 °C. Thereafter, the ZF embryos were dechorionated using sharp forceps and pre-selected for GFP protein expression using an M165 FC fully apochromatic corrected stereo microscope (Leica, Wetzlar, Germany). Dechorionated ZF embryos were maintained in an E3 solution with PTU (final concentration of 75 µM), to avoid pigmentation formation.

#### 2.2.10. pDNA Fluorescence Labeling for In Vivo Assays

pND1 labeling was performed using Ulysis™ Alexa Fluor™ 594 Nucleic Acid Labeling Kit (Molecular Probes, Eugene, OR, USA). 1/10 volume of 3 M sodium acetate (pH 5.2) and 2 volumes of absolute ethanol were added to 1 µg of pDN1, placed at −80 °C for 30 min, and then centrifuged for 15 min at 12,000 rpm to precipitate the plasmid. The pellet was washed with 70% ethanol and allowed to air dry. The pellet was resuspended with 20 µL of labeling buffer (provided in the kit) and then 1 µL of Alexa594 dye solution was added. Labeling buffer was used until 25 µL of final volume was reached. The reaction was incubated at 80 °C for 15 min and at the end of this time, the reaction was stopped by placing the tubes on ice. The tubes were centrifuged with a MiniSpin to redeposit all contents at the bottom of the tube and the labeled pND1 was stored at 4 °C.

#### 2.2.11. Confocal Microscopy

To evaluate the transfection efficiency and internalization capacity of the MTS–CPP/pND1 systems in ZF embryos, the confocal microscopy technique was used. For this study, ZF embryos that expressed the fluorescent protein GFP were previously selected. For each condition, 6 zebrafish embryos were transferred in a glass chamber slide (LabTek, 4-chamber slide, Sigma-Aldrich) in a volume of 200 µL E3 + PTU. Each type of nanoparticle was formulated in 50 µL as described above using Alexa594-labelled pND1 (1 µg, final concentration) and added to the ZF embryos. After 24 h of transfection, ZF embryos were washed 3 times with PBS and fixed with 4% PFA in PBS for 20 min at room temperature with stirring. ZF embryos were washed 5 times with PBS and imaged by confocal microscopy on an inverted Zeiss LSM800 microscope using a 10×/0.30 lens. All confocal acquisitions were performed using a 488 nm diode laser with the specific GFP filter (486 nm–561 nm) and a 561 nm diode laser with a specific Alexa594 filter (592 nm–614 nm). A total of 15 images per sample were acquired using z-stack mode with a z-stack interval of 3 µm. Acquired images were analyzed using ImageJ software (Version 1.54). Z-projection for each sample was performed by summing fluorescence intensities to one image.

#### 2.2.12. Toxicity Test on Zebrafish Embryos

The test to evaluate toxicity was carried out 48 h after incubation of zebrafish embryos with the different nanoparticles under study. For each condition, 6 zebrafish embryos were transferred in a glass chamber slide (LabTek, 4-chamber slide, Sigma-Aldrich) in a volume of 200 µL E3 + PTU. Each type of nanoparticle was formulated in 50 µL as described above using 3 different pDNA amounts (1 µg, 2 µg, and 5 µg, final concentration) and added to the ZF embryos. After 48 h incubation at 28 °C, ZF embryos were anesthetized with 0.02% Tricaine solution and imaged using an M165 FC fully apochromatic corrected stereo microscope (Leica, Wetzlar, Germany). All ZF embryos were analyzed in terms of their development and morphology. The size of each ZF embryo was subsequently determined using ImageJ software.

#### 2.2.13. Statistical Analysis

Data are presented as mean ± standard error of the mean. Statistically significant differences were evaluated by one-way analysis of variance (ANOVA), with Bonferroni’s multiple comparison test. Data were analyzed using GraphPad Prisma software, V9.0.0 (GraphPad Software Inc., New York, NY, USA). A *p*-value of <0.05 was considered statistically significant.

## 3. Results and Discussion

### 3.1. Effect of PEGylation on Nanocomplexes Formulation

CPPs have been widely explored for the delivery of therapeutic genetic material. Due to their physicochemical properties, it is possible to formulate stable nanocomplexes with some of the CPPs that protect nucleic acids until their delivery to target cells [47,48,49]. In previous articles, it was demonstrated that the addition of the MTS sequence into certain CPPs, namely WRAP1 (W1) and WRAP5 (W5) peptides, conferred specific targeting to the mitochondria, enabling the delivery of a mitochondrial gene into the target organelle [29,38]. In this work, we evaluated the effect of incorporating a polyethylene-glycol 2000 (PEG) moiety into these nanocarriers, to form more stable delivery systems compatible with an in vivo delivery such as in zebrafish (ZF) embryos (described here) or, later, in other animal models. Although PEG has recently been shown to be an immunogenic molecule in certain circumstances, its incorporation into delivery systems has been widely explored. PEG has been used successfully, improving the therapeutic efficacy of various delivery systems, namely liposome-based nanoparticles and PEGylated lipid nanoparticles (LNPs) in the field of mRNA-based vaccines [40,50].

The peptide nanocomplexes were formulated using the most suitable N/P ratio described in previous works (N/P ratio = 5 in water) [29,38] in which various percentages of PEG–CPPs were tested in the complexes formulation (5%, 10%, and 20%). The results for the size and polydispersity index (PdI) of PEG–nanocomplexes are presented in Table 1. WRAP1-based complexes with 5% PEG display sizes around 200 nm, while with 20% PEG, the sizes are around 160 nm, with no significant changes when compared to the results obtained for WRAP1 systems without PEG (*p* = 0.9933). In the case of WRAP5 nanocomplexes with 5 to 20% PEG, the sizes vary from 114 nm to 99 nm on average, and there is a statistically significant change in relation to the sizes of the WRAP5/pND1 systems without PEG (*** *p* < 0.001), which have an average size of 186 nm. 

The PEG–MTS–WRAP1/pND1 vectors exhibit sizes around 60 nm for the three analyzed PEG percentages in the formulation, with no significant changes in terms of size when comparing the three different formulations to each other (*p* ≥ 0.05). However, there is a significant size reduction (**** *p* < 0.0001) compared to the MTS–WRAP1/pND1 systems without PEG. The MTS–WRAP5/pND1 systems previously presented sizes of around 175 nm. The addition of PEG only significantly influenced the size of the nanoparticles when 20% PEG was added (*** *p* < 0.001), reducing the average size to values below 100 nm. The addition of 5 and 10% PEG did not cause significant changes in the average size of these systems (*p* = 0.9955 and *p* = 0.7934, respectively).

Although there are some changes in complex sizes with the addition of PEG, all nanocomplexes have sizes below 200 nm and PdI remains below 0.4, demonstrating that the systems are monodisperse and that they exhibit ideal physicochemical properties to be further evaluated by in vitro and in vivo studies.

### 3.2. Effect of PEGylation on Nanocomplexes Stability

The stability of PEG–peptide nanocomplexes was evaluated 24 h and 7 days after formulation. The results are summarized in Table 2. In detail, the complexes remained stable during the first 24 h, with no major fluctuations in terms of size. The PEG–WRAP1 nanocomplexes with 5% PEG maintained their size after 24 h of formulation (*p* = 0.9912), while those with 10% and 20% had a significant increase in size (**** *p* < 0.0001), which may indicate some thickness of the complexes over time resulting in particle growth. For the PEG–WRAP5 nanocomplexes, there was an increase in the sizes of those formulated with 5% PEG (*** *p* < 0.001) and for those with 10% and 20%, the sizes remained identical (*p* = 0.9581 and *p* = 0.1533, respectively). In contrast, the MTS-containing complexes (PEG–MTS–WRAP1 and PEG–MTS–WRAP5) showed average sizes after 24 h very similar to the sizes obtained after formulation. The PdI values indicated no loss of homogeneity during the first 24 h, with values remaining between 0.1 and 0.4. 

After 7 days, the PEG–WRAP1 and PEG–WRAP5 did not prove to be stable, displaying a dramatic increase in their average sizes to values above 300 nm (**** *p* < 0.0001). The loss of stability of these nanocomplexes was also reflected in the PdI parameter, with values higher than 0.4, even reaching 1. These PdI values indicate that the complexes are polydisperse and lose their stability over time. It is reasonable to assume that the complexes experienced aggregation into larger particles over time, and after 7 days, they may cluster and form macroscopic aggregates. In contrast, the nanocomplexes containing the MTS sequence presented stability in terms of sizes, with no major changes compared to the values at the time of formulation. The sizes for the MTS–PEG–WRAP1 complexes remained around 60 nm for the three considered percentages of PEG (all *p*-values ≥ 0.05) after 7 days. The MTS–PEG–WRAP5 complexes increased their average size slightly after 7 days, namely from 167 nm to 291 nm (** *p* < 0.01) for complexes with 10% PEG and from 93 nm to 197 nm for those with 20% PEG (*** *p* < 0.001). However, this size increases at 7 days post-formulation of the nanocomplexes and does not preclude their use since the sizes remain below 300 nm. 

### 3.3. Peptide-Based Nanocomplexes Are Stable in Saline Solution

According to the results presented in Table 1 and Table 2, we found adequate evidence to focus our investigation on the WRAP complexes bearing 20% PEG, using these to perform the subsequent tests. As the purpose of the current study was, mainly, to test the developed peptide nanocomplexes on the zebrafish (ZF) model, we then evaluated the maintenance of the sizes and PdI of the complexes after resuspending in the saline E3 solution (zebrafish medium). These measurements aimed to verify the stability of the nanocomplexes in the ZF medium since the presence of salts can interfere with the electrostatic interaction between the peptides and pND1. The results for PEG–WRAP/pND1 or PEG–MTS–WRAP:pND1 were compared to previously analyzed non-PEGylated nanocomplexes such as (KH)_9_/pDNA and CpMTP/pDNA[REF]. 

The results presented in the Supplementary Material (SM), Table S2**,** demonstrate that all the analyzed CPP/pND1 complexes exhibited sizes of around 100 nm–250 nm, except for CpMTP/pND1, which presented sizes of around 350 nm. The same trend is reflected through the PdI values, showing that all nanocomplexes are homogeneous when resuspended in an E3 solution (PdI values between 0.2 and 0.4). Compared to the values in Table 1, the sizes of the PEGylated nanocomplexes are significantly larger (*** *p* < 0.001) when they are in contact with E3 medium; however, this increase does not compromise the physicochemical properties of the complexes as the sizes remained below 275 nm. In the case of the non-PEGylated complexes, there was a slight increase in sizes for the CpMTP/pND1 systems (*** *p* < 0.001) when they were in E3 medium and a decrease in sizes in the (KH)_9_ peptide-based systems (**** *p* < 0.0001) in E3 medium, compared to the sizes previously obtained in water [29]. Therefore, we can conclude that except for CpMTP/pND1 (size over 250 nm), all analyzed peptide-based complexes under study are stable when resuspended with an E3 solution, maintaining their size and homogeneity.

### 3.4. In Vitro Biocompatibility of Peptide-Based Nanocomplexes

**Figure 1:**
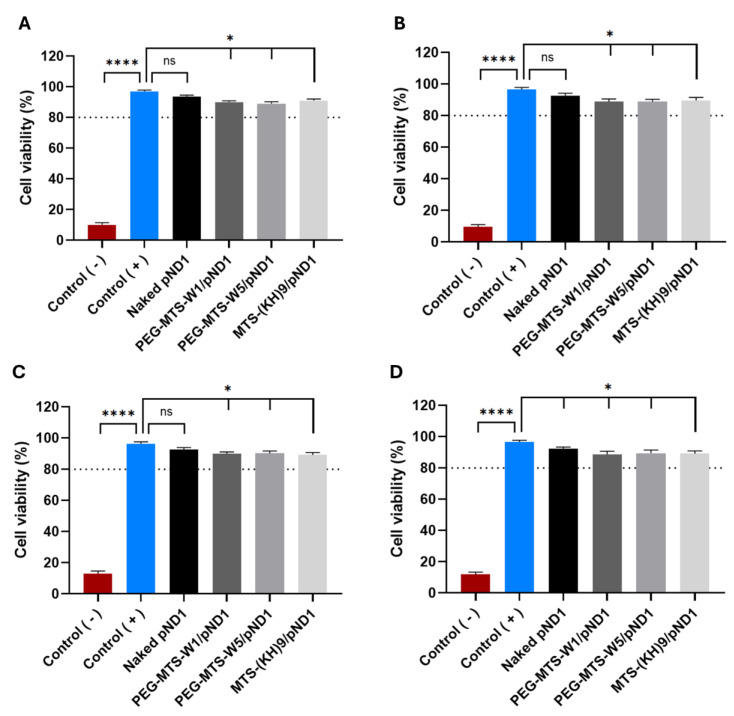
Cellular viability of human astrocyte cells ((**A**) 24 h, (**B**) 48 h) and lung smooth muscle cells ((**C**) 24 h, (**D**) 48 h) after incubation with naked pND1 and the 20% PEG–MTS–WRAP1/pND1 (PEG–MTS–W1/pND1), 20% PEG–MTS–WRAP5/pND1 (PEG–MTS–W5/pND1) and MTS–(KH)_9_/pND1 nanocomplexes formulated at N/P ratio of 5 (pND1 = 1 µg). Non-transfected cells were used as a positive control (Control (+)) and cells treated with ethanol were used as a negative control (Control (−)). Data were analyzed by one-way ANOVA with Bonferroni’s multiple comparison test (ns—non-significant (*p* > 0.05); * *p* ˂ 0,05; **** *p* ˂ 0.0001).

The cytotoxicity of the PEG/MTS–CPP/pND1 nanocomplexes was evaluated using the colorimetric method of the MTT assay. Toxicity was assessed in two distinct cell lines, namely human astrocyte cell line and lung smooth muscle cells. Cell viability (%) was determined after 24 h and 48 h of cell incubation with the PEG/MTS–CPP/pND1 complexes (0.1 µg of pND1 per well), formulated at an N/P ratio of 5. Non-transfected cells were used as a positive control and cells treated with ethanol were considered the negative control. The cytotoxicity results for these two types of cells are presented in the graphs of Figure 1. The results of cellular viability of human astrocyte cells for 24 h and 48 h are shown in the graphs in Figure 1A and Figure 1B, respectively. Cell viability of human astrocytes transfected with the three different nanocomplexes is greater than 80% after 24 and 48 h. The viability of these cells was around 90% after 24 h when incubated with the PEG–MTS–WRAP1/pND1 nanocomplexes and 88.8% after 48 h. For the PEG–MTS–WRAP5/pND1 complexes, the viability of human astrocytes was 89% after 24 h and 88.4% after 48 h. 

In comparison, cells incubated with the MTS–(KH)_9_/pND1 complexes showed a cell viability of 91% after 24 h and 89.7% after 48 h, similar to the WRAP complexes. The viability data in human astrocyte cells demonstrated that the three PEG/MTS–CPP/pND1 systems are not cytotoxic in this cell line, since cell viabilities are equal to or greater than 80%—an indication of the non-cytotoxic profile of compounds according to ISO 10993-5 [51,52].

The cellular viability results in lung smooth muscle cells are also presented in Figure 1 (Figure 1C for 24 h and Figure 1D for 48 h). The results are very similar to those obtained in astrocytes. In lung smooth muscle cells transfected with the PEG–MTS–WRAP1/pND1 nanocomplexes, cell viability was 89.8% at 24 h and 88.6% at 48 h. In the case of cells incubated with PEG–MTS–WRAP5/pND1 complexes, viability at 24 h was 90.2% and at 48 h it was 89.3%. The viability for cells incubated with the MTS–(KH)_9_/pND1 systems was 89.1% after 24 h and 88.9% after 48 h. 

The results in Figure 1 demonstrate that the developed peptide-based nanocomplexes are biocompatible and do not present significant cytotoxicity in the two cell lines studied. Therefore, these nanocomplexes can be considered secure and convenient delivery nanosystems to be further researched toward mitochondrial gene delivery approaches.

### 3.5. PEG/MTS–CPP/pND1 Nanocomplexes Do Not Cause Hemolysis

**Figure 2:**
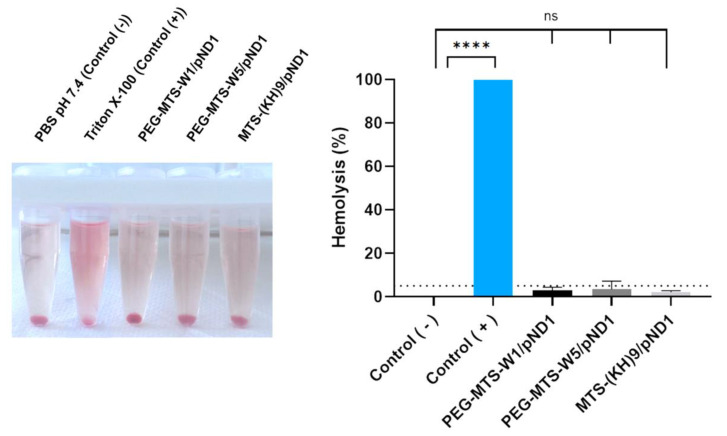
In vitro hemolysis assay using rat red blood cells (RBCs), which were incubated with 20% PEG–MTS–WRAP1/pND1, 20% PEG–MTS–WRAP5/pND1, and MTS–(KH)_9_/pND1 (1 µg of pND1, N/P ratio = 5). The negative control was incubated with PBS pH 7.4, while in the positive control, RBCs were incubated with Triton X-100 (1%) to provoke hemolysis. The hemolysis percentages were calculated according to Formula (1). Data are presented as mean (%) ± SD (n = 3). Data were analyzed by one-way ANOVA with Bonferroni’s multiple comparison test (ns—non-significant (*p* > 0.05); **** *p* ˂ 0.0001).

A hemolysis assay was carried out to consolidate the biocompatibility of these systems before moving on to toxicity tests in in vivo models. For this purpose, rat blood was used, wherein, RBCs were collected and subsequently incubated with the delivery systems. Two control groups were considered, a negative control in which PBS pH 7.4 was added and a positive control in which Triton X-100 was added. Triton X-100 detergent was chosen as a positive control as it was revealed in a previous study to be the best compound to cause hemolysis with more stable and reproducible results [53]. The hemolysis rate results are shown in Figure 2. For nanosystems to be considered non-hemolytic and to be used in medical applications, according to the ISO/TR 7406 standard, their hemolysis rate must be less than 5% [54]. The two PEG–MTS–CPP/pND1 nanocomplexes presented very low hemolysis rates (2.30% for PEG–MTS–WRAP1/pND1 and 3.75% for PEG–MTS–WRAP5/pND1). In comparison, the MTS–(KH)_9_/pND1 nanocomplex has a hemolysis rate of 1.25%. Using the one-way ANOVA test, we verified that there is no statistically significant difference between the hemolysis percentages of RBCs incubated with the three types of system when compared to the negative control (RBCs incubated with PBS). The hemolysis percentages indicated that the PEG/MTS peptide-based nanocomplexes under study have good blood biocompatibility, a very promising result concerning the biosafety of these complexes for in vivo applications. 

### 3.6. Mitochondria Targeting Capacity of PEG/MTS–CPP/pND1 Complexes 

**Figure 3:**
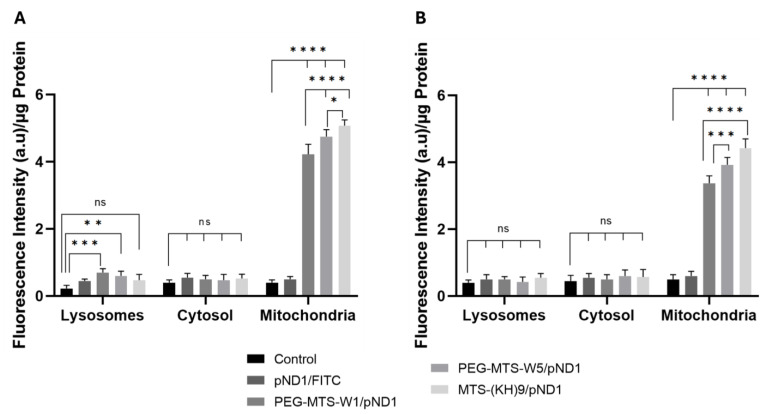
Quantification of FITC fluorescence intensity ((a.u)/µg Protein) in the lysosomes, cytosol, and mitochondria of human astrocyte cells (**A**) and lung smooth muscle cells (**B**), after 24 h of transfection with 20% PEG–MTS–WRAP1/pND1 (PEG–MTS–W1/pND1), 20% PEG–MTS–WRAP5/pND1 (PEG–MTS–W5/pND1) and MTS–(KH)_9_/pND1 systems. All complexes were formulated with an N/P ratio = 5 (pND1 = 1 µg). Untreated cells and naked pND1 stained with FITC were used as controls. Data were analyzed by two-way ANOVA with Bonferroni’s multiple comparison test (ns—non-significant (*p* > 0.05); * *p* ˂ 0.05; ** *p* ˂ 0.01; *** *p* ˂ 0.001; **** *p* ˂ 0.0001).

The cellular uptake of the developed PEG–MTS–WRAP/pND1–FITC complexes and their accumulation into different cellular organelles have been evaluated by monitoring the organelle-associated FITC fluorescence, 24 h after transfection mediated by these carriers. Non-transfected cells were used as control. Figure 3 summarizes the obtained data for human astrocytes (Figure 3A) and lung smooth muscle cells (Figure 3B). The results demonstrated the cellular uptake of the conceived peptide nanocomplexes into both cells; however, with FITC fluorescence levels being detected to a very different extent depending on the organelle. For both cells, a strong accumulation of all the PEG–MTS–WRAP/pND1–FITC complexes into the mitochondria was observed compared with the correspondent accumulation of the complexes into the lysosomes or the cytosol (for all cases: **** *p* ˂ 0.0001). For astrocytes, the FITC fluorescence intensity in the lysosomes was, however, statistically significant in comparison with the control cells for the transfection mediated by both PEG–MTS–WRAP1/pND1–FITC, *** *p* ˂ 0.001, and PEG–MTS–WRAP5/pND1–FITC, ** *p* ˂ 0.01 (Figure 3A). This indicates that a minor amount of WRAP1- and WRAP5-based complex accumulation into the lysosomes of astrocytes cannot be excluded. On the contrary, for these cells, the FITC fluorescence detected in the cytosol showed no statistically significant differences (ns) in comparison with the control cells.

Concerning the MTS–(KH)_9_/pND1 complex, no FITC fluorescence accumulation is observed in the lysosomes or the cytosol but a significantly higher accumulation is observed in the mitochondria compared to that observed with PEG–MTS–WRAP/pND1.

For lung muscle cells, the FITC fluorescence intensity in both lysosomes and cytosol was comparable to the FITC levels found in the control cells and no statistically significant differences (ns) were found (Figure 3B). For both cells, it becomes clear that peptide-based nanocomplexes are internalized by the cells and efficiently target mitochondria, with variations in the FITC fluorescence intensity between the three studied carriers. The mitochondria accumulation increased in the order MTS–(KH)_9_/pND1 > PEG–MTS–WRAP5/pND1 > PEG–MTS–WRAP1/pND1 complexes. However, in some cases the differences were low (please consult the statistical analysis available in Figure 3), which may reflect the different transfection efficiency displayed by the nanocomplexes, which may consequently influence the subsequent process of protein expression into mitochondria.

### 3.7. Peptide-Based Complexes Increase ND1 Levels In Vitro

**Figure 4:**
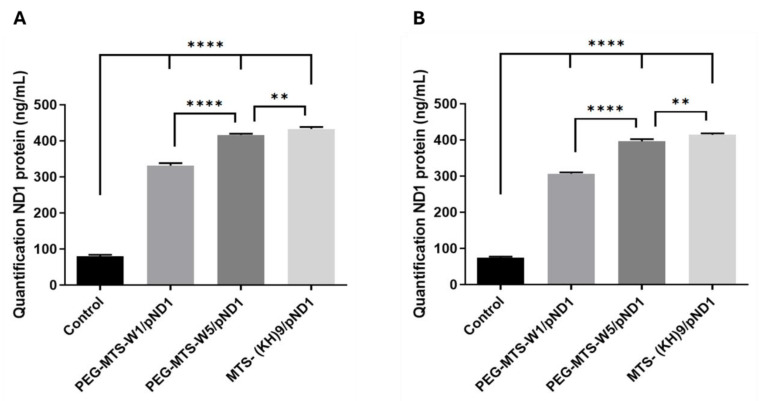
Quantification of ND1 protein levels (ng/mL) in human astrocyte cells (**A**) and lung smooth muscle cells (**B**), after 48 h of transfection with 20% PEG–MTS–WRAP1/pND1 (PEG–MTS–W1/pND1), 20% PEG–MTS–WRAP5/pND1 (PEG–MTS–W5/pND1), and MTS–(KH)_9_/pND1 systems (pND1 = 1 µg for all). All complexes were formulated with an N/P ratio = 5. Data were analyzed by one-way ANOVA with Bonferroni’s multiple comparison tests (** *p* = 0.0041 (**A**) and 0.0015 (**B**),**** *p* ˂ 0.0001).

The demonstrated mitochondria-targeting ability of the developed PEG/MTS–CPP/pND1 complexes enriches their value as carriers for gene delivery into this organelle. To confirm the potential for mitochondrial protein expression, the ND1 protein levels after 48 h of transfection of astrocytes and lung muscle cells mediated by these complexes were determined. As explained in the experimental section, in this assay, an Elisa Kit was employed, and non-transfected cells were considered as control. The results are presented in Figure 4. ND1 is an endogenous gene, and, therefore, this fact can explain the ND1 protein levels found in the control cells. The content of this mitochondrial protein can be considerably increased when both cells are transfected with the studied PEG/MTS–peptide/pND1 nanocomplexes. As observed in Figure 4A,B, there are statistically significant differences between each of the complexes and the control astrocytes and muscle cells: for both cells, **** *p* < 0.0001. This revealed the capacity of the conceived nanocomplexes to target mitochondria, release into this organelle the genetic carried content and, ultimately, produce the correspondent mitochondrial ND1 protein. The ND1 protein is a subunit of NADH dehydrogenase, which is located in the mitochondrial inner membrane and is the largest of the five complexes of the electron transport chain.

In detail, among the formulated complexes, and for both cells, PEG–MTS–WRAP1/pND1 was the system leading to the lowest produced ND1 content (**** *p* < 0.0001 for PEG–MTS–WRAP1/pND1 versus PEG–MTS–WRAP5/pND1, Figure 4). However, for astrocytes and muscle cells, the MTS–(KH)_9_/pND1 complexes were the complexes leading to the superior ND1 protein levels compared to both PEG–MTS–WRAP/pND1 complexes. These observations can be correlated with the different transfection efficiency (Figure 3) displayed by the complexes, with implications in the extent of ND1 protein expression. The results presented in this work agree well with previous research focused on the transfection behavior of PEG-free CPP-based complexes, where ND1 protein levels were monitored in both HeLa and fibroblast cells [38].

### 3.8. Peptide Nanocomplexes Efficiently Internalize in Zebrafish Embryos

After confirming that peptide-based nanocomplexes possess physicochemical properties suitable for cellular transfection, are biocompatible to cells, stable in both water and E3 saline solution, target mitochondria, and are able of ND1 protein expression, the next step was to evaluate their transfection efficiency and internalization capacity in a dechorionated zebrafish embryo in vivo model. The choice of ZF to carry out these tests is, mainly, because this in vivo model is easy to reproduce, with a small size, and a large number of descendants [37,55]. Furthermore, the fact that it is possible to obtain optically transparent embryos is essential to be able to visualize and analyze the internalization of the delivery systems that are intended to be tested [56]. The use of embryos has advantages compared to the use of adult ZF since it is considered that the embryos do not feel pain or other types of discomfort [57].

To evaluate the internalization of the developed peptide nanocomplexes, a confocal microscopy study was conducted. To adequately use this technique, pND1 was labeled with an Alexa-594 dye (pND1–Alexa594) before complex formulation. To evaluate the labeling of pND1 and monitor the transfection capacity of the MTS–CPP/pND1–Alexa594 complexes, a small test was carried out on HEK293T-GFP cells before moving to studies with ZF. HEK293T-GFP cells were incubated with pND1–Alexa594 and with the MTS–WRAP1/pND1–Alexa594 complexes. The results of incubation in HEK293T-GFP cells are presented in Supplementary Materials, Figure S1. From the analysis of Figure S1, we can observe that the incubation of pND1 labeled with Alexa594 in cells (Figure S1B) only resulted in a few points of plasmid accumulation (red signal). However, these points of pND1 accumulation are located outside the cells, with no overlapping of the pND1 fluorescence with the fluorescence of the GFP protein (green signal) present in the cytoplasm nor with the fluorescence of the cell nucleus labeling with DAPI (blue signal). In the case of HEK293T-GFP cells incubated with the MTS–WRAP1/pND1–Alexa594 complexes (Figure S1C), there were also points of accumulation of pND1 (red signal), however, the fluorescent signal is mainly located within the cytoplasm of the cells. The accumulation of pND1 inside the cells reveals that the MTS–WRAP1/pND1–Alexa594 complexes were able to transfect and internalize in these cells and that the labeling of pND1 with the Alexa594 probe allows, through confocal microscopy, effective visualization of the location and distribution of these nanocomplexes. Moreover, to confirm that PEG–MTS–CPP/pND1 complexes target the mitochondria of HEK293T cells and mediate gene/protein expression, ND1 protein levels were quantified after transfection of these cells with the developed carriers. The results for ND1 content can be consulted in Supplementary Materials, Figure S2. For the transfection mediated by the developed complexes, high ND1 levels were obtained in comparison with the control cells (**** *p* < 0.0001). This proves not only the mitochondria targeting skill exhibited by the MTS–CPP/pND1 complexes but also their successful role in promoting ND1 protein expression.

**Figure 5:**
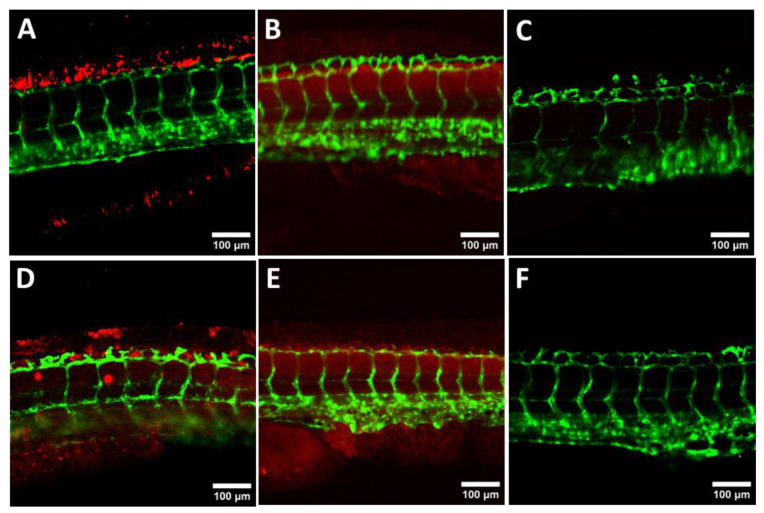
Evaluation of the ability of CPP-based nanocomplexes for ZF embryo transfection. Representative confocal images of ZF embryos expressing the GFP protein (green signal) transfected with different CPP-based complexes encapsulating Alexa594-labelled pND1. (**A**) 20% PEG–MTS–WRAP1/pND1 with 2 µg, (**B**) 20% PEG–MTS–WRAP1/pND1 with 1 µg, (**C**) 20% PEG–MTS–WRAP1/pND1 with 0.5 µg, (**D**) 20% PEG–WRAP5/pND1 with 2 µg, and (**E**) MTS–(KH)_9_/pND1 with 2 µg imaged after 24 h incubation. Peptide nanocomplexes were formulated at an N/P ratio of 5 using the indicated final plasmid concentrations. Untransfected ZF embryos were used as control (**F**). Bars represent 100 µm.

After verifying that the labeling of pND1 with the Alexa594 probe makes it possible to monitor and confirm the internalization and mitochondria targeting ability of the nanocomplexes, studies to evaluate the transfection and internalization capacity of the peptide-based complexes in ZF embryos were performed. For a better visualization, GFP-expressing ZF embryos were selected. Using the confocal microscopy technique, images of the embryos were obtained 24 h after transfection with the nanocomplexes under study. The results of transfection and internalization of the PEG–MTS–WRAP1/pND1 and PEG–MTS–WRAP5/pND1 compared to MTS–(KH)_9_/pND1 nanocomplexes are shown in Figure 5. The images in Figure 5A–C demonstrated that the 20% PEG–MTS–WRAP1/pND1 complexes were able to internalize in zebrafish embryos in a dose-dependent manner. At 2 µg of pND1–Alexa594, we observed a dotted accumulation of red dots, especially on the dorsal fin of the ZF embryo. In contrast, at 1 µg, a more homogeneous and diffuse distribution throughout the embryo body was observed, whereas at 0.5 µg, no more red fluorescence was shown. For the 20% PEG–MTS–WRAP5/pND1 complexes, at 2 µg, we observed a combination of dots and homogeneous distribution through the ZF embryos (Figure 5D). The internalization of MTS-(KH)_9_/pND1 nanocomplexes (Figure 5E) revealed a homogeneous and diffuse distribution at 2 µg comparable to that observed for 20% MTS–PEG–WRAP1/pND1 at 1 µg.

All tested MTS–CPP complexes demonstrated the ability to internalize in zebrafish embryos using 2–1 µg of pND1, resulting in a nearly homogeneous distribution throughout the body of the embryo compared to non-treated ZF embryos (Figure 5F). These results demonstrated the ability of these nanocomplexes to transfect into ZF embryos, highlighting their value for effective in vivo gene delivery. This will potentially contribute to advance in vivo research in the mitochondrial gene therapy field.

### 3.9. Toxicity Evaluation In Vivo Zebrafish Embryo Model

**Figure 6:**
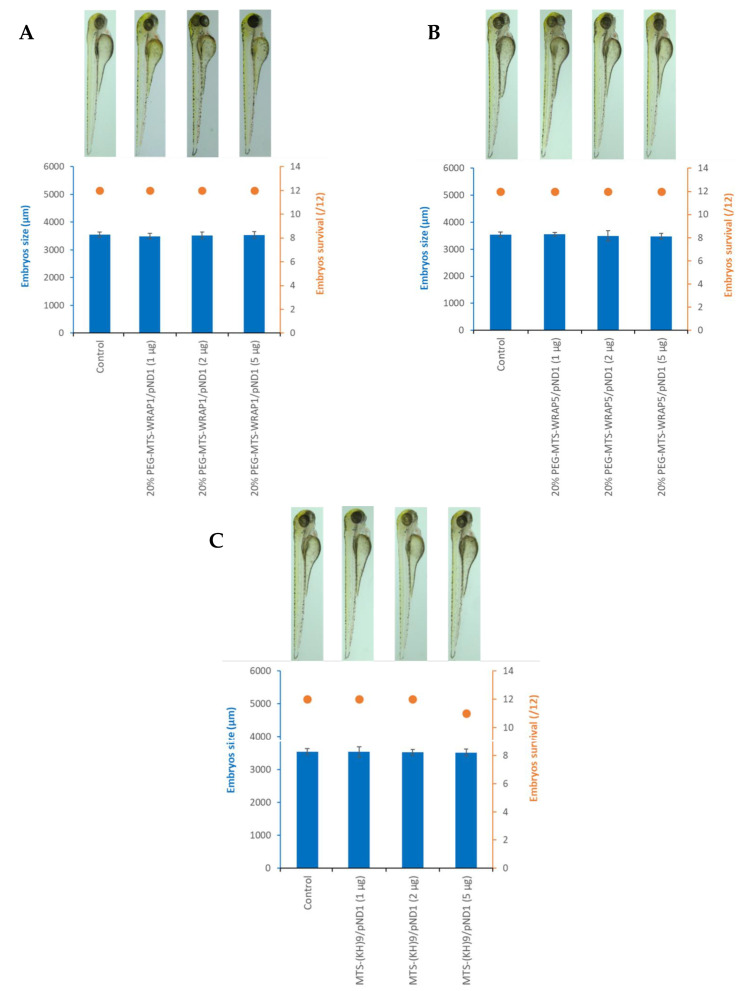
Assessment of the toxicity of the 20% PEG–MTS–WRAP1/pND1 (**A**), 20% PEG–MTS–WRAP5/pND1 (**B**), and MTS–(KH)_9_/pND1 (**C**) nanocomplexes (N/P ratio = 5) in ZF embryos. Toxicity was assessed through the average size of the embryos (µm) and their survival (/12) after 48 h of incubation. Non-transfected embryos were used as a control group. All nanocomplexes were tested at three different amounts (1, 2, and 5 µg).

After verifying that the PEG–MTS–WRAP/pND1 and MTS–(KH)_9_/pND1 complexes can successfully transfect and internalize into ZF embryos, we wanted to evaluate whether these delivery systems cause any toxicity in these in vivo models, particularly in terms of embryo development/growth and mortality. For this purpose, three quantities of nanocomplexes were tested for each type of system under study, namely 1, 2, and 5 µg, which corresponds to the amount of pND1 used to formulate the nanocomplexes. The toxicity of the peptide complexes was analyzed 48 h after incubation in ZF embryos. The results of development/growth and mortality of ZF embryos are shown in Figure 6.

The toxicity results of the 20% PEG–WRAP1/pND1 complexes (without MTS sequence) are presented in Figure S3A (available in Supplementary Materials). Embryos transfected with the 20% PEG–WRAP1/pDN1 nanocomplexes did not show changes in development/growth in relation to the non-transfected control group. No deaths occurred for the two lowest amounts of the complexes (1 and 2 µg). However, in the group of embryos transfected with 5 µg of 20% PEG–WRAP1/pDN1 complexes, 10 out of 12 embryos survived.

The toxicity of the WRAP1-based complexes was tested in the ZF embryo in vivo model. Figure 6A presents the obtained results for the 20% PEG–MTS–WRAP1/pND1 nanocomplexes (for 1, 2, and 5 µg pDNA). Embryos transfected with the 20% PEG–MTS–WRAP1/pND1 complexes exhibited a survival rate of 100% and their development/growth was identical to the control group (non-transfected embryos). For the three quantities of considered complexes, there were no changes in the morphology of the embryos or their development, with sizes around 3550 µm, similar to embryos that were not transfected. The data in Figure 6A suggested that the 20% PEG–MTS–WRAP1/pND1 nanocomplexes are stable and do not present associated toxicity in ZF embryos. 

The toxicity of the WRAP5-based nanocomplexes was also analyzed. The results for the 20% PEG–WRAP5/pND1 nanocomplexes (without MTS sequence) are shown in Figure S3B, in the Supplementary Materials. The results show that the 20% PEG–WRAP5/pDN1 nanocomplexes do not present any toxicity to ZF embryos in the three pDNA quantities under study. In the three groups of ZF embryos, no associated deaths were recorded, and the development/growth of the embryos was similar to the control group. We can, therefore, conclude that the 20% PEG–WRAP5/pND1 nanocomplexes are stable and do not cause toxicity in ZF embryos.

Figure 6B presents the toxicity results of the 20% PEG–MTS–WRAP5/pND1 nanocomplexes in ZF embryos. The 20% PEG–MTS–WRAP5/pND1 complexes were demonstrated to be stable and non-toxic to this in vivo model since all embryos survived in the three pDNA quantities used and there were no changes in their development/growth. 

The toxicity data of the MTS-(KH)_9_/pND1 nanocomplexes are represented in Figure 6C. As with the PEG-MTS-WRAP/pND1 complexes, the MTS–(KH)_9_/pND1 systems do not present associated toxicity when transfected and internalized in ZF embryos. Embryos incubated with these nanocomplexes presented a development and growth profile identical to embryos in the control group for the three pDNA quantities tested. The survival rate for 1 and 2 µg of MTS–(KH)_9_/pND1 nanocomplexes is 100%, with only 1 embryo in 12 dying for the amount of 5 µg of MTS–(KH)_9_/pND1 complexes.

The toxicity of the CpMTP/pND1 nanocomplexes was also tested in ZF embryos, as shown in Figure S4. The CpMTP/pND1 complexes did not reveal any toxic effects for ZF embryos in the three different pDNA amounts used to transfect these in vivo models. The embryos from these three groups demonstrated growth and development similar to the control group, with no associated deaths. 

The tested peptide-based nanocomplexes were demonstrated to not elicit toxicity in zebrafish embryos, not causing changes in their morphology or growth/development, nor causing deaths associated with their use in this in vivo model. Therefore, the developed peptide delivery systems were revealed to be safe, stable, and biocompatible, holding great promise as carriers for in vivo mitochondrial gene transfection.

## 4. Conclusions

Diseases originating from mitochondrial dysfunction caused by mutations in mtDNA continue to lack therapies that enable treatment. Mitochondrial gene therapy is a very promising approach, but it is deeply dependent on a safe, biocompatible, and efficient vector to deliver therapeutic genetic material. Although MTS–CPP nanocomplexes had already demonstrated auspicious results in vitro, their in vivo evaluation was missing, retarding potential clinical translation. In this work, MTS–WRAP/pND1 complexes were PEGylated, and the formed PEG–nanocomplexes displayed adequate sizes, are stable up to 7 days after formulation, biocompatible to astrocytes and lung smooth muscle cells, and hemocompatible. The PEG–MTS–WRAP/pND1 complexes exhibited mitochondria targeting ability and promoted mitochondrial protein production. Following this, in vivo research was conducted in ZF embryos to determine the toxic profile of MTS–CPP-based systems—mandatory for biomedical applications—and their capacity for in vivo transfection. The ZF in vivo model was revealed to be a very useful testing platform to assess nanocomplexes toxicity, filling the distance between in vitro and rodent models. 

Compared to the unPEGylated CpMTP/pND1 nanocomplex, we observed the formation of bigger nanoparticles (~350 nm) in the E3 solution used as an aqueous solution to care for ZF embryos. This could impact their application to ZF assays. In contrast, the other analyzed unPEGylated MTS–(KH)_9_/pND1 complex was revealed to have slightly smaller nanocomplexes (~60 nm) and to have a more pronounced mitochondrial accumulation and ND1 protein expression compared to both PEG–MTS–WRAP/pND1 nanocomplexes. This phenomenon could be explained either by the smaller particle size (=better internalization of MTS–(KH)_9_/pND1) or by the fact that PEGylation could mask the nanocomplexes (=lower internalization of PEG–MTS–WARP/pND1).

PEGylation of lipid-based nanoparticles has proved particularly efficient in conferring longer systemic circulation, improving their pharmacokinetics and efficiency [58]. Also for peptide-based nanocomplexes, PEGylation was applied for the same reasons as shown for PEG–RICK:siRNA [59], for PEG–PepFect14:pDNA [60], and for PEG–NicFect55:pDNA [61]. In all cases, PEGylation improved the in vivo activity of the delivered nucleic acids (siRNA or pDNA). We observed the same improvement of nanocomplex stability in saline solution (E3 solution of the ZF embryos) by adding a PEG moiety to the WRAP nanocomplexes (see Table 2 and Table S2). Unexpectedly, MTS–(KH)_9_ nanocomplexes showed good stability even in saline solutions without adding any PEG entity. The results presented here do not reveal the exact reason for this stability. The only visible difference is the absence of tryptophan in the CPP (KH)_9_ sequence. We can only speculate that a possible Π-cation (tryptophan/NaCl) interaction could modify the hydration properties of tryptophan-containing CPPs, making them more sensitive to saline solutions. Further experiments are required to determine whether tryptophan can be sensitive to saline conditions.

The results obtained in ZF embryos demonstrated that the developed MTS–peptide nanocomplexes are stable, that they can internalize and distribute themselves throughout zebrafish, and that they do not present toxicity, nor do they cause malformations or changes in the normal growth and development of ZF. These very promising results demonstrate the biocompatibility and high performance of the developed MTS–(KH)_9_/pND1 nanocomplexes and with slightly less activity also for the PEG/MTS–WRAP/pND1 nanocomplexes in a living system, opening the path for advances in mitochondrial gene therapy in vivo research.

## Figures and Tables

**Table 1:** Average size and PdI of peptide-based nanocomplexes at N/P = 5 after formulation in water.

**WRAP1 Systems**		
**Systems**	**Average Size (nm)**	**PdI**
WRAP1/pND1 [29]	161 ± 9	0.300 ± 0.023
5% PEG–WRAP1/pND1	197 ± 26	0.446 ± 0.089
10% PEG–WRAP1/pND1	124 ± 7	0.083 ± 0.129
20% PEG–WRAP1/pND1	160 ± 11	0.346 ± 0.061
**WRAP5 Systems**		
**Systems**	**Average Size (nm)**	**PdI**
WRAP5/pND1 [29]	186 ± 10	0.320 ± 0.030
5% PEG–WRAP5/pND1	114 ± 9	0.245 ± 0.064
10% PEG–WRAP5/pND1	101 ± 15	0.263 ± 0.036
20% PEG–WRAP5/pND1	99 ± 5	0.222 ± 0.061
**MTS–WRAP1 Systems**		
**Systems**	**Average Size (nm)**	**PdI**
MTS–WRAP1/pND1 [29]	197 ± 8	0.200 ± 0.020
5% PEG–MTS–WRAP1/pND1	58 ± 7	0.190 ± 0.016
10% PEG–MTS–WRAP1/pND1	57 ± 3	0.144 ± 0.013
20% PEG–MTS–WRAP1/pND1	65 ± 8	0.158 ± 0.020
**MTS–WRAP5 Systems**		
**Systems**	**Average Size (nm)**	**PdI**
MTS–WRAP5/pND1 [29]	175 ± 11	0.320 ± 0.030
5% PEG–MTS–WRAP5/pND1	173 ± 14	0.377 ± 0.049
10% PEG–MTS–WRAP5/pND1	167 ± 19	0.321 ± 0.034
20% PEG–MTS–WRAP5/pND1	93 ± 15	0.167 ± 0.086

Notes: Explanation of the formulations: 5% PEG–WRAP = 5% PEG–WRAP + 95% WRAP; 10% PEG–WRAP = 10% PEG–WRAP + 90% WRAP; 20% PEG–WRAP = 20% PEG–WRAP + 80% WRAP; 5% PEG–MTS–WRAP = 5% PEG–WRAP + 47.5% MTS–WRAP + 47.5% WRAP; 10% PEG–MTS–WRAP = 10% PEG–WRAP + 45% MTS–WRAP + 45% WRAP; 20% PEG–MTS–WRAP = 20% PEG–WRAP + 40% MTS–WRAP + 40% WRAP. All nanocomplexes were formulated at an N/P of 5, in water with 1 µg pND1. The values were determined with the data calculated from the acquisition of three independent experiments. Data were analyzed by one-way ANOVA followed by the Bonferroni test, *p* ˂ 0.05 was considered statistically significant.

**Table 2:** Average size and PdI of peptide-based nanocomplexes formulated at N/P ratio = 5 measured at 24 h and at 7 days after formulation.

**WRAP1 Systems**				
**Systems**	**After 24 h**		**After 7 Days**	
	**Mean Size (nm)**	**PdI**	**Mean Size (nm)**	**PdI**
5% PEG–WRAP1/pND1	203 ± 9	0.402 ± 0.029	695 ± 53	0.592 ± 0.194
10% PEG–WRAP1/pND1	260 ± 15	0.346 ± 0.040	3161 ± 197	1.000 ± 0.000
20% PEG–WRAP1/pND1	448 ± 27	0.457 ± 0.115	978 ± 30	0.956 ± 0.028
**WRAP5 Systems**				
**Systems**	**After 24 h**		**After 7 Days**	
	**Mean Size (nm)**	**PdI**	**Mean Size (nm)**	**PdI**
5% PEG–WRAP5/pND1	233 ± 14	0.336 ± 0.093	320 ± 18	0.233 ± 0.055
10% PEG–WRAP5/pND1	96 ± 4	0.288 ± 0.045	1640 ± 143	1.000 ± 0.092
20% PEG–WRAP5/pND1	114 ± 10	0.252 ± 0.082	309 ± 22	0.409 ± 0.058
**MTS-WRAP1 Systems**				
**Systems**	**After 24 h**		**After 7 Days**	
	**Mean SIZE (nm)**	**PdI**	**Mean Size (nm)**	**PdI**
5% PEG–MTS–WRAP1/pND1	67 ± 5	0.211 ± 0.063	62 ± 17	0.215 ± 0.039
10% PEG–MTS–WRAP1/pND1	60 ± 9	0.161 ± 0.028	60 ± 11	0.174 ± 0.085
20% PEG–MTS–WRAP1/pND1	65 ± 11	0.136 ± 0.041	64 ± 14	0.134 ± 0.044
**MTS-WRAP5 Systems**				
**Systems**	**After 24 h**		**AFTER 7 Days**	
	**Mean Size (nm)**	**PdI**	**Mean Size (nm)**	**PdI**
5% PEG–MTS–WRAP5/pND1	82 ± 6	0.473 ± 0.158	85 ± 27	0.515 ± 0.100
10% PEG–MTS–WRAP5/pND1	253 ± 21	0.338 ± 0.099	291 ± 21	0.722 ± 0.167
20% PEG–MTS–WRAP5/pND1	73 ± 2	0.155 ± 0.071	197 ± 18	0.329 ± 0.072

Notes: Explanation of the formulations: 5% PEG–WRAP = 5% PEG–WRAP + 95% WRAP; 10% PEG–WRAP = 10% PEG–WRAP + 90% WRAP; 20% PEG–WRAP = 20% PEG–WRAP + 80% WRAP; 5% PEG–MTS–WRAP = 5% PEG–WRAP + 47.5% MTS–WRAP + 47.5% WRAP; 10% PEG–MTS–WRAP = 10% PEG–WRAP + 45% MTS–WRAP + 45% WRAP; 20% PEG–MTS–WRAP = 20% PEG–WRAP + 40% MTS–WRAP + 40% WRAP. All nanocomplexes were formulated at an N/P of 5, in water with 1 µg pND1. The values were determined with the data calculated from three independent assays. Data were analyzed by one-way ANOVA followed by the Bonferroni test, *p* ˂ 0.05 was considered statistically significant.

## Data Availability

All research data is included in manuscript and Supplementary Materials Files.

## Supplementary Materials

The following supporting information can be downloaded at: https://www.mdpi.com/article/10.3390/pharmaceutics16070961/s1, Table S1: List of the synthesized peptides and MTS sequence, including information on peptide sequence, total residues, isotopic mass, and positive charges. Table S2: Average size and PdI of peptide-based nanocomplexes formulated at N/P ratio of 5 resuspended in E3 solution. Figure S1: Confocal images of non-treated HEK293T-GFP cells (**A**), HEK293T-GFP cells incubated with pND1 labeled with Alexa594 (pND1–Alexa594) (**B**), and HEK293T-GFP cells transfected with MTS–WRAP1/pND1–Alexa594 nanoparticles (**C**). Figure S2: Quantification of ND1 protein levels in HEK293T cells after transfection with the developed MTS–CPP/pND1 complexes. Figure S3: Assessment of the toxicity of the 20% PEG–WRAP1/pND1 (**A**) and 20% PEG–WRAP5/pND1 (**B**) systems (N/P ratio of 5) in ZF embryos. Toxicity was assessed through the average size of the embryos (µm) and their survival (/12) after 48 h of incubation. Non-transfected embryos were used as a control group. Both systems were tested at 3 different amounts of pDNA (1 µg, 2 µg, and 5 µg). Figure S4: Assessment of the toxicity of CpMTP/pND1 systems (N/P ratio of 5) in ZF embryos. Toxicity was assessed through the average size of the embryos (µm) and their survival (/12) after 48 h of incubation. Non-transfected embryos were used as a control group. The CpMTP/pND1 systems were tested at 3 different amounts of pDNA (1 µg, 2 µg, and 5 µg).

## References

[1] Marchi S., Guilbaud E., Tait S.W.G., Yamazaki T., Galluzzi L. (2022). Mitochondrial control of inflammation. Nat. Rev. Immunol..

[2] Popov L.D. (2020). Mitochondrial biogenesis: An update. J. Cell. Mol. Med..

[3] Kadenbach B. (2021). Complex IV—The regulatory center of mitochondrial oxidative phosphorylation. Mitochondrion.

[4] Singh A.P., Salvatori R., Aftab W., Kohler A., Carlstrom A., Forne I., Imhof A., Ott M. (2020). Molecular Connectivity of Mitochondrial Gene Expression and OXPHOS Biogenesis. Mol. Cell.

[5] Fontecilla-Camps J.C. (2021). Primordial bioenergy sources: The two facets of adenosine triphosphate. J. Inorg. Biochem..

[6] Schlieben L.D., Prokisch H. (2020). The Dimensions of Primary Mitochondrial Disorders. Front. Cell Dev. Biol..

[7] Kaniak-Golik A., Skoneczna A. (2015). Mitochondria-nucleus network for genome stability. Free Radic. Biol. Med..

[8] Zardoya R. (2020). Recent advances in understanding mitochondrial genome diversity. F1000Research.

[9] Stoccoro A., Coppedè F. (2021). Mitochondrial DNA Methylation and Human Diseases. Int. J. Mol. Sci..

[10] Nissanka N., Moraes C.T. (2020). Mitochondrial DNA heteroplasmy in disease and targeted nuclease-based therapeutic approaches. EMBO Rep..

[11] Lawless C., Greaves L., Reeve A.K., Turnbull D.M., Vincent A.E. (2020). The rise and rise of mitochondrial DNA mutations. Open Biol..

[12] La Morgia C., Maresca A., Caporali L., Valentino M.L., Carelli V. (2020). Mitochondrial diseases in adults. J. Intern. Med..

[13] Gusic M., Prokisch H. (2021). Genetic basis of mitochondrial diseases. FEBS Lett..

[14] Kopinski P.K., Singh L.N., Zhang S., Lott M.T., Wallace D.C. (2021). Mitochondrial DNA variation and cancer. Nat. Rev. Cancer.

[15] Kumar R., Harilal S., Parambi D.G.T., Kanthlal S.K., Rahman M.A., Alexiou A., Batiha G.E.-S., Mathew B. (2022). The Role of Mitochondrial Genes in Neurodegenerative Disorders. Curr. Neuropharmacol..

[16] Zhang L., Zhang Z., Khan A., Zheng H., Yuan C., Jiang H. (2020). Advances in drug therapy for mitochondrial diseases. Ann. Transl. Med..

[17] Tinker R.J., Lim A.Z., Stefanetti R.J., McFarland R. (2021). Current and Emerging Clinical Treatment in Mitochondrial Disease. Mol. Diagn. Ther..

[18] Aravintha Siva M., Mahalakshmi R., Bhakta-Guha D., Guha G. (2019). Gene therapy for the mitochondrial genome: Purging mutations, pacifying ailments. Mitochondrion.

[19] Faria R., Boisguérin P., Sousa Â., Costa D. (2023). Delivery Systems for Mitochondrial Gene Therapy: A Review. Pharmaceutics.

[20] Falabella M., Minczuk M., Hanna M.G., Viscomi C., Pitceathly R.D.S. (2022). Gene therapy for primary mitochondrial diseases: Experimental advances and clinical challenges. Nat. Rev. Neurol..

[21] Yoshinaga N., Numata K. (2022). Rational Designs at the Forefront of Mitochondria-Targeted Gene Delivery: Recent Progress and Future Perspectives. ACS Biomater. Sci. Eng..

[22] Buchke S., Sharma M., Bora A., Relekar M., Bhanu P., Kumar J. (2022). Mitochondria-Targeted, Nanoparticle-Based Drug-Delivery Systems: Therapeutics for Mitochondrial Disorders. Life.

[23] Nam S.H., Park J., Koo H. (2023). Recent advances in selective and targeted drug/gene delivery systems using cell-penetrating peptides. Arch. Pharmacal Res..

[24] Sun Y., Zhang H., Lu G., Wang H., Lu Y., Fan L. (2023). Mitochondria-targeted cancer therapy based on functional peptides. Chin. Chem. Lett..

[25] Taylor R.E., Zahid M. (2020). Cell Penetrating Peptides, Novel Vectors for Gene Therapy. Pharmaceutics.

[26] Konate K., Dussot M., Aldrian G., Vaissiere A., Viguier V., Neira I.F., Couillaud F., Vives E., Boisguerin P., Deshayes S. (2019). Peptide-Based Nanoparticles to Rapidly and Efficiently “Wrap ’n Roll” siRNA into Cells. Bioconjug. Chem..

[27] Abe N., Fujita S., Miyamoto T., Tsuchiya K., Numata K. (2023). Plant Mitochondrial-Targeted Gene Delivery by Peptide/DNA Micelles Quantitatively Surface-Modified with Mitochondrial Targeting and Membrane-Penetrating Peptides. Biomacromolecules.

[28] Yilmaz N., Kodama Y., Numata K. (2021). Lipid Membrane Interaction of Peptide/DNA Complexes Designed for Gene Delivery. Langmuir.

[29] Faria R., Vives E., Boisguerin P., Sousa A., Costa D. (2021). Development of Peptide-Based Nanoparticles for Mitochondrial Plasmid DNA Delivery. Polymers.

[30] McGrath P., Li C.-Q. (2008). Zebrafish: A predictive model for assessing drug-induced toxicity. Drug Discov. Today.

[31] Saleem S., Kannan R.R. (2018). Zebrafish: An emerging real-time model system to study Alzheimer’s disease and neurospecific drug discovery. Cell Death Discov..

[32] Teame T., Zhang Z., Ran C., Zhang H., Yang Y., Ding Q., Xie M., Gao C., Ye Y., Duan M. (2019). The use of zebrafish (Danio rerio) as biomedical models. Anim. Front..

[33] Zhang K., Liang J., Brun N.R., Zhao Y., Werdich A.A. (2021). Rapid Zebrafish Behavioral Profiling Assay Accelerates the Identification of Environmental Neurodevelopmental Toxicants. Environ. Sci. Technol..

[34] Haque E., Ward A. (2018). Zebrafish as a Model to Evaluate Nanoparticle Toxicity. Nanomaterials.

[35] Patton E.E., Zon L.I., Langenau D.M. (2021). Zebrafish disease models in drug discovery: From preclinical modelling to clinical trials. Nat. Rev. Drug Discov..

[36] Bhattarai P., Turgutalp B., Kizil C. (2022). Zebrafish as an Experimental and Preclinical Model for Alzheimer’s Disease. ACS Chem. Neurosci..

[37] Shen C., Zuo Z. (2020). Zebrafish (Danio rerio) as an excellent vertebrate model for the development, reproductive, cardiovascular, and neural and ocular development toxicity study of hazardous chemicals. Environ. Sci. Pollut. Res..

[38] Faria R., Paul M., Biswas S., Vives E., Boisguerin P., Sousa A., Costa D. (2022). Peptides vs. Polymers: Searching for the Most Efficient Delivery System for Mitochondrial Gene Therapy. Pharmaceutics.

[39] Shi D., Beasock D., Fessler A., Szebeni J., Ljubimova J.Y., Afonin K.A., Dobrovolskaia M.A. (2022). To PEGylate or not to PEGylate: Immunological properties of nanomedicine’s most popular component, polyethylene glycol and its alternatives. Adv. Drug Deliv. Rev..

[40] Ibrahim M., Ramadan E., Elsadek N.E., Emam S.E., Shimizu T., Ando H., Ishima Y., Elgarhy O.H., Sarhan H.A., Hussein A.K. (2022). Polyethylene glycol (PEG): The nature, immunogenicity, and role in the hypersensitivity of PEGylated products. J. Control Release.

[41] Yadav D., Dewangan H.K. (2021). PEGYLATION: An important approach for novel drug delivery system. J. Biomater. Sci. Polym. Ed..

[42] Harris J.M., Chess R.B. (2003). Effect of pegylation on pharmaceuticals. Nat. Rev. Drug Discov..

[43] Elsadek N.E., Abu Lila A.S., Ishida T. (2020). Immunological responses to PEGylated proteins. Polymer-Protein Conjugates.

[44] Faria R., Albuquerque T., Neves A.R., Bhatt H., Biswas S., Cardoso A.M., Pedroso de Lima M.C., Jurado A.S., Costa D. (2020). Physicochemical characterization and targeting performance of triphenylphosphonium nano-polyplexes. J. Mol. Liq..

[45] Costa D., Costa C., Caldeira M., Cortes L., Queiroz J.A., Cruz C. (2017). Targeting of Cellular Organelles by Fluorescent Plasmid DNA Nanoparticles. Biomacromolecules.

[46] Lawson N.D., Weinstein B.M. (2002). In vivo imaging of embryonic vascular development using transgenic zebrafish. Dev. Biol..

[47] Xie J., Bi Y., Zhang H., Dong S., Teng L., Lee R.J., Yang Z. (2020). Cell-Penetrating Peptides in Diagnosis and Treatment of Human Diseases: From Preclinical Research to Clinical Application. Front. Pharmacol..

[48] Subia B., Reinisalo M., Dey N., Tavakoli S., Subrizi A., Ganguli M., Ruponen M. (2019). Nucleic acid delivery to differentiated retinal pigment epithelial cells using cell-penetrating peptide as a carrier. Eur. J. Pharm. Biopharm..

[49] Boisguerin P., Deshayes S., Gait M.J., O’Donovan L., Godfrey C., Betts C.A., Wood M.J., Lebleu B. (2015). Delivery of therapeutic oligonucleotides with cell penetrating peptides. Adv. Drug Deliv. Rev..

[50] d’Avanzo N., Celia C., Barone A., Carafa M., Di Marzio L., Santos H.A., Fresta M. (2020). Immunogenicity of Polyethylene Glycol Based Nanomedicines: Mechanisms, Clinical Implications and Systematic Approach. Adv. Ther..

[51] Lopez-Garcia J., Lehocky M., Humpolicek P., Saha P. (2014). HaCaT Keratinocytes Response on Antimicrobial Atelocollagen Substrates: Extent of Cytotoxicity, Cell Viability and Proliferation. J. Funct. Biomater..

[52] Hao M., Liu R. (2019). Molecular mechanism of CAT and SOD activity change under MPA-CdTe quantum dots induced oxidative stress in the mouse primary hepatocytes. Spectrochim. Acta A Mol. Biomol. Spectrosc..

[53] Saebo I.P., Bjoras M., Franzyk H., Helgesen E., Booth J.A. (2023). Optimization of the Hemolysis Assay for the Assessment of Cytotoxicity. Int. J. Mol. Sci..

[54] Niza E., Nieto-Jimenez C., Noblejas-Lopez M.D.M., Bravo I., Castro-Osma J.A., Cruz-Martinez F., Buchaca M.M.S., Posadas I., Canales-Vazquez J., Lara-Sanchez A. (2019). Poly(Cyclohexene Phthalate) Nanoparticles for Controlled Dasatinib Delivery in Breast Cancer Therapy. Nanomaterials.

[55] Dubey A., Ghosh N.S., Singh R. (2022). Zebrafish as An Emerging Model: An Important Testing Platform for Biomedical Science. J. Pharm. Negat. Results.

[56] Parkkila S., Parikka M., Hammaren M.M., Aspatwar A. (2019). Rapid Evaluation of Toxicity of Chemical Compounds Using Zebrafish Embryos. J. Vis. Exp..

[57] Wiley D.S., Redfield S.E., Zon L.I. (2017). Chemical screening in zebrafish for novel biological and therapeutic discovery. The Zebrafish: Disease Models and Chemical Screens.

[58] Tenchov R., Sasso J.M., Zhou Q.A. (2023). PEGylated Lipid Nanoparticle Formulations: Immunological Safety and Efficiency Perspective. Bioconjug. Chem..

[59] Aldrian G., Vaissiere A., Konate K., Seisel Q., Vives E., Fernandez F., Viguier V., Genevois C., Couillaud F., Demene H. (2017). PEGylation rate influences peptide-based nanoparticles mediated siRNA delivery in vitro and in vivo. J. Control Release.

[60] Veiman K.L., Kunnapuu K., Lehto T., Kiisholts K., Parn K., Langel U., Kurrikoff K. (2015). PEG shielded MMP sensitive CPPs for efficient and tumor specific gene delivery in vivo. J. Control Release.

[61] Freimann K., Arukuusk P., Kurrikoff K., Vasconcelos L.D.F., Veiman K.L., Uusna J., Margus H., Garcia-Sosa A.T., Pooga M., Langel U. (2016). Optimization of in vivo DNA delivery with NickFect peptide vectors. J. Control Release.

