# Supplementary material for: Upgrading Mitochondria-Targeting Peptide-Based Nanocomplexes for Zebrafish In Vivo Compatibility Assays
Source: Pharmaceutics. 2024 Jul 20;16(7):961. doi: 10.3390/pharmaceutics16070961 (PMC11281276; doi:10.3390/pharmaceutics16070961)
Supplement: Supplementary file 1 [file pharmaceutics-16-00961-s001.zip › pharmaceutics-3061967-supplementary.pdf]

Supplementary Material

Upgrading Mitochondria-Targeting Peptide-Based Nanocomplexes for Zebrafish In Vivo Compatibility Assays

Rúben Faria, Eric Vivès, Prisca Boisguérin, Simon Descamps, Ângela Sousa and Diana Costa

Table S1 - List of the synthesized peptides and MTS sequence, including information on peptide sequence, total residues, isotopic mass, and positive charges.

Peptide	Peptide sequence	Total residues	Isotopic Mass [g/mol]	Positive Charges
MTS	NH2-MLSLRQSIRFFK-CONH2	12	1523.88	3
WRAP1	NH2-LLWRLWRLLWRLWRLL-CONH2	16	2290.42	5
WRAP5	NH2-LLRLLRWWWRLLRLL-CONH2	15	2104.34	5
(KH)9	NH2-KHKHKHKHKHKHKHKHKH-CONH2	18	2403.41	9
CpMTP	NH2-ARLLWLLRGLTLGTAPRRA-CONH2	19	2132.32	4
MTS-WRAP1	NH2-MLSLRQSIRFFK-LLWRLWRLLWRLWRLL-CONH2	28	3797.27	8
MTS-WRAP5	NH2-MLSLRQSIRFFK-LLRLLRWWWRLLRLL-CONH2	27	3611.19	8
MTS-(KH)9	NH2-MLSLRQSIRFFK-KHKHKHKHKHKHKHKHKH-CONH2	30	3910.26	12

Results

Table S2 – Average size and PdI of peptide-based nanocomplexes formulated at N/P ratio of 5 resuspended in E3 solution.

Systems	Mean Size (nm)	PdI
20% PEG-WRAP1/pND1	101 ± 10	0.268 ± 0.033
20% PEG-WRAP5/pND1	187 ± 6	0.474 ± 0.091
20% PEG-MTS-WRAP1/pND1	240 ± 14	0.354 ± 0.069
20% PEG-MTS-WRAP5/pND1	275 ± 29	0.217 ± 0.025
(KH)9/pND1	127 ± 4	0.215 ± 0.064
MTS-(KH)9/pND1	57 ± 9	0.206 ± 0.015
CpMTP/pND1	347 ± 13	0.342 0.072

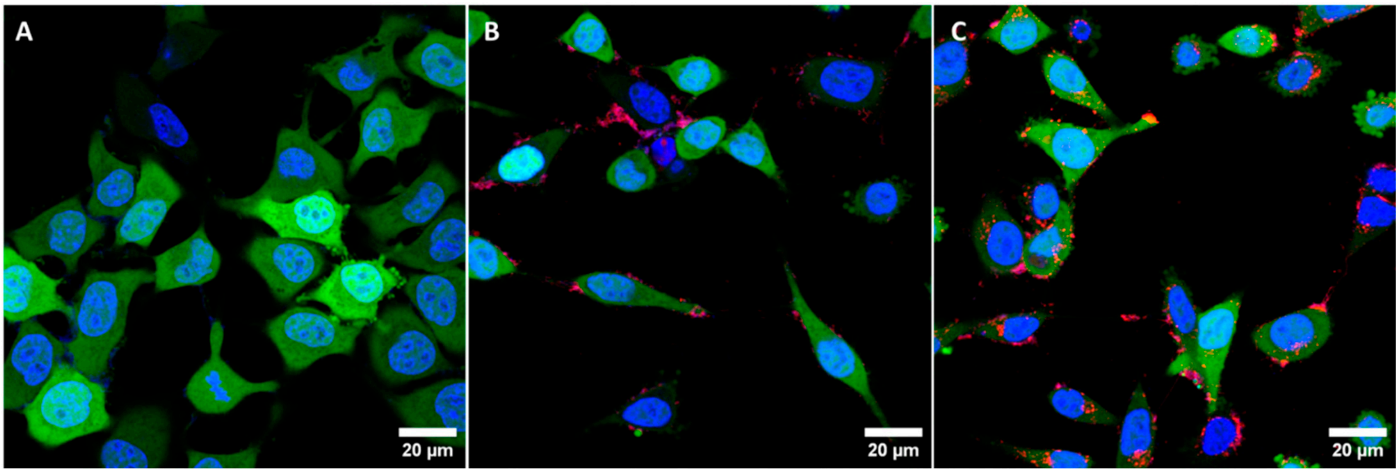

Figure S1. Confocal images of non-treated HEK293T-GFP cells (**A**), HEK293T-GFP cells incubated with pND1 labeled with Alexa594 (pND1-Alexa594) (**B**) and HEK293T-GFP cells transfected with MTS-WRAP1/pND1-Alexa594 nanoparticles (**C**). The blue signal corresponds to cell nuclei labeled with Hoechst 33342, the green signal to the GFP protein produced intrinsically in HEK cells, and the red signal corresponds to the Alexa594 fluorescence of pND1. Images were obtained 2 h after cell transfection, using a 40x objective, lasers: 488 - 1.5%/700V; 405 - 1.0%/700V; 561 – 1.0%/650V.

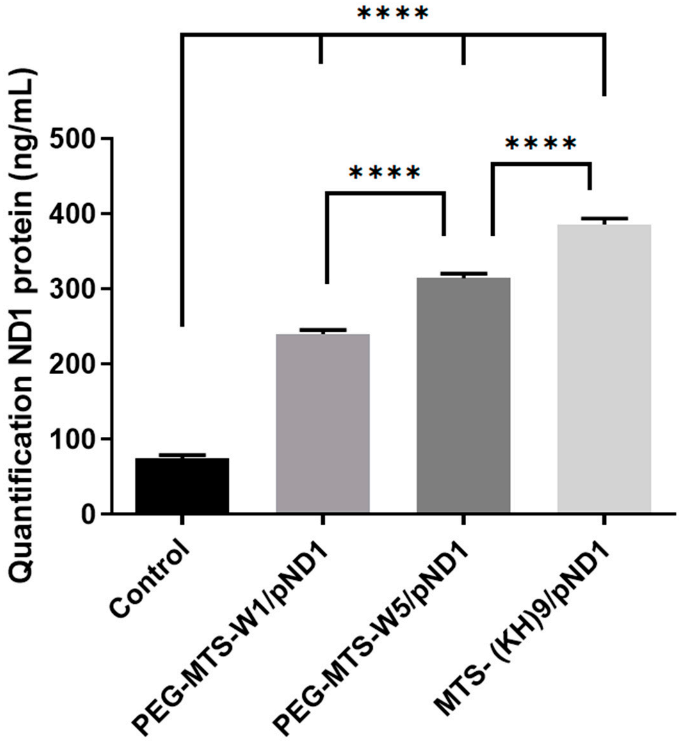

Figure S2. Quantification of ND1 protein levels (ng/mL) in HEK293T cells, after 48 h of transfection with 20% PEG-MTS-WRAP1/pND1 (PEG-MTS-W1/pND1), 20% PEG-MTS-WRAP5/pND1 (PEG-MTS-W5/pND1) and MTS-(KH)9/pND1 systems. All complexes were formulated with the N/P ratio = 5. Data were analyzed by one-way ANOVA with Bonferroni's multiple comparison test.

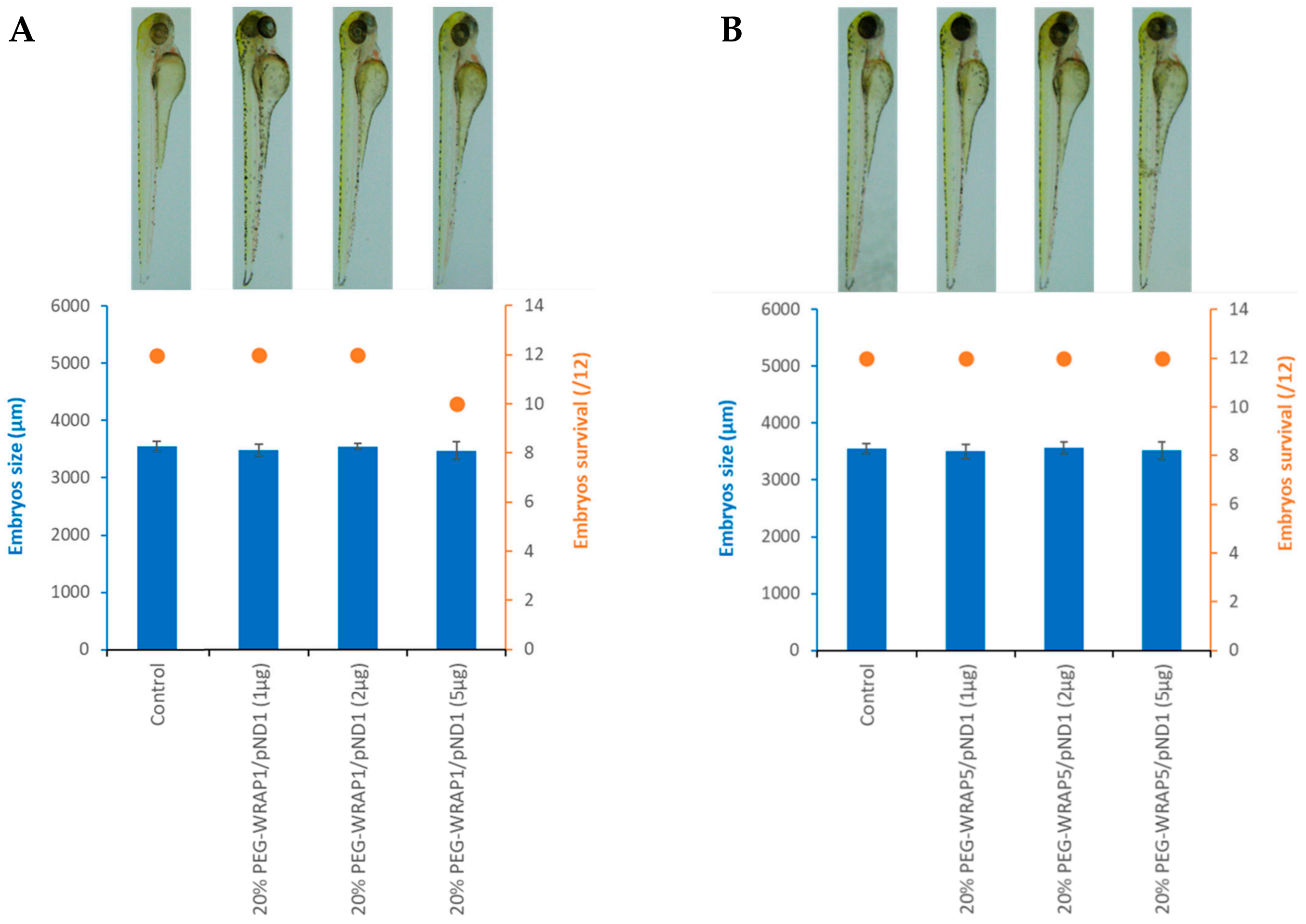

Figure S3. Assessment of the toxicity of the 20% PEG-WRAP1/pND1 (**A**) and 20% PEG-WRAP5/pND1 (**B**) systems (N/P ratio of 5) in ZF embryos. Toxicity was assessed through the average size of the embryos (µm) and their survival (/12) after 48 h of incubation. Non-transfected embryos were used as a control group. Both systems were tested at 3 different amounts of pDNA (1 µg, 2 µg, and 5 µg).

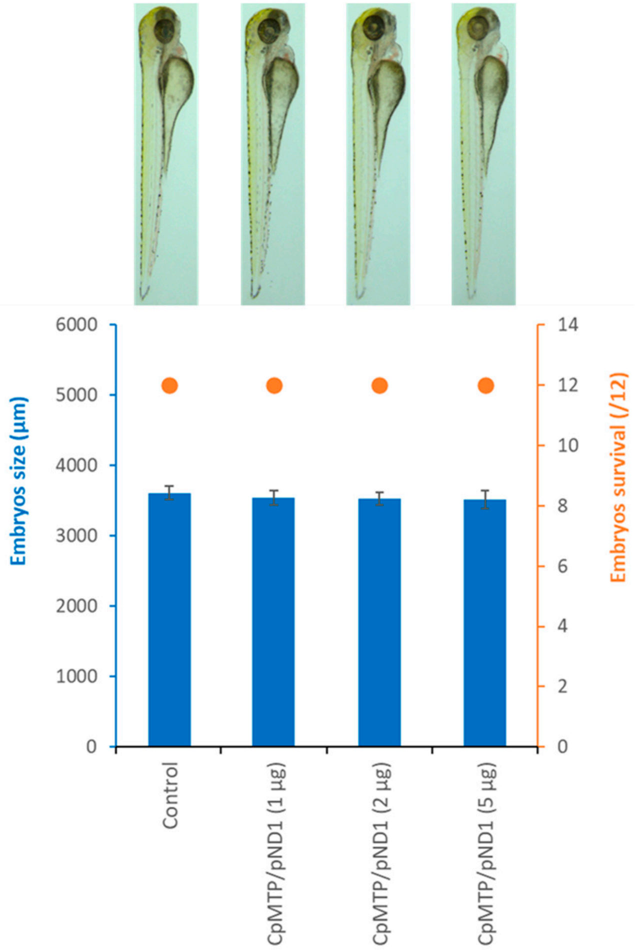

Figure S4. Assessment of the toxicity of CpMTP/pND1 systems (N/P ratio of 5) in ZF embryos. Toxicity was assessed through the average size of the embryos (µm) and their survival (/12) after 48 h of incubation. Non-transfected embryos were used as a control group. The CpMTP/pND1 systems were tested at 3 different amounts of pDNA (1 µg, 2 µg, and 5 µg).
